# Human sperm cooperate to transit highly viscous regions on the competitive pathway to fertilization

**DOI:** 10.1038/s42003-023-04875-2

**Published:** 2023-05-06

**Authors:** Sa Xiao, Jason Riordon, Alex Lagunov, Mahta Ghaffarzadeh, Thomas Hannam, Reza Nosrati, David Sinton

**Affiliations:** 1 Department of Mechanical and Industrial Engineering, University of Toronto, Toronto, ON M5S 3G8 Canada; 2 CCRM IVF Network, Lone Tree, CO 80124 USA; 3 CCRM Toronto, Toronto, ON M4W 3R2 Canada; 4 Department of Mechanical and Aerospace Engineering, Monash University, Clayton, VIC 3800 Australia

**Keywords:** Collective cell migration, Cellular motility

## Abstract

Human sperm compete for fertilization. Here, we find that human sperm, unexpectedly, cooperate under conditions mimicking the viscosity contrasts in the female reproductive tract. Sperm attach at the head region to migrate as a cooperative group upon transit into and through a high viscosity medium (15-100 cP) from low viscosity seminal fluid. Sperm groups benefit from higher swimming velocity, exceeding that of individual sperm by over 50%. We find that sperm associated with a group possess high DNA integrity (7% fragmentation index) – a stark contrast to individual sperm exhibiting low DNA integrity (> 50% fragmentation index) – and feature membrane decapacitation factors that mediate sperm attachment to form the group. Cooperative behaviour becomes less prevalent upon capacitation and groups tend to disband as the surrounding viscosity reduces. When sperm from different male sources are present, related sperm preferentially form groups and achieve greater swimming velocity, while unrelated sperm are slowed by their involvement in a group. These findings reveal cooperation as a selective mode of human sperm motion – sperm with high DNA integrity cooperate to transit the highly viscous regions in the female tract and outcompete rival sperm for fertilization – and provide insight into cooperation-based sperm selection strategies for assisted reproduction.

## Abstract (web summary)

Human sperm are shown to cooperate upon transit into high viscosity media with related sperm displaying higher tendencies to cooperate, suggesting sperm attachment is preferential and confers a benefit under inter-ejaculate conditions

## Introduction

The competitive nature of sperm is prevalent in mammalian fertilization, including human fertilization^1–3^. Genetic analyses reveal that the rapid evolution of genes involved in human spermatogenesis follows a similar evolutionary trajectory to that of most mammals experiencing sperm competition, such as chimpanzees^4,5^. This competition, whereby sperm from either intra-ejaculate (same male) or inter-ejaculate (multiple males) compete for fertilization within the female reproductive tract, can enable evolutionary adaptations in sperm physiology, morphology, and behavior^1,6,7^.

The fertilization success of an individual sperm is predicated on its ability to outperform competitors and reach the ovum while maintaining proper physical and biochemical function^6–8^. Sperm motility, DNA integrity, and plasma membrane integrity are key measures of the fertilization ability of sperm and are positively correlated to fertilization success in natural and assisted reproduction^9–14^. Human sperm, and in general mammalian sperm, have developed various behaviors that lead to increased motility and more precise navigation to the ovum, which also signify the presence of high DNA and membrane integrity. Human and bull sperm, which exhibit a three-dimensional rolling waveform in bulk fluid, can transition to a two-dimensional planar swimming mode that enables faster migration through the viscous conditions of the female tract^15^. Human and bull sperm display boundary-following swimming behavior, in a similar manner to that of microorganisms such as *Escherichia coli*^16^, which enables navigation via the anatomical microfolds lining the female tract to the site of fertilization^17,18^. Human and mouse sperm exhibit rheotaxis, in which the sperm with a rolling waveform can reorient and swim against the opposing flow of secretions to progress toward the ovum^19,20^. These types of swimming modes are typically exemplified by sperm with high DNA integrity^21–23^.

In rare events, competition can motivate cooperative behavior, as observed in select beetles^24^, fishflies^25^, echidnas^26^, opossums^27^, and muroid rodents^28^. These sperm cooperate by physically attaching, generally at the sperm head via morphological or membrane interactions, and forming groups that can consist of a few to hundreds of sperm^29,30^. For instance, sperm of muroid rodents feature a falciform morphology, including an apical hook, and sperm attach via the hook to form groups of hundreds of sperm^28,31–33^. The flagella of sperm associated with a group are typically unbound, and can beat in asynchrony^24,26,28^, synchrony^25^, or opposite synchrony as exemplified in the opossum sperm pair^27^. It has also been shown among mouse sperm exhibiting the rolling waveform that attachment can prevent rolling and thereby promote a more linear swimming path and a faster rate of progression^34^.

Cooperation has been observed to facilitate higher swimming velocity in most cases^25,27,29,32^. There can also be preferential cooperation, in which sperm groups form based on relatedness among sperm in the presence of inter-ejaculate conditions, as found in the promiscuous deer mouse (*Peromyscus maniculatus*)^33^. Taken together, these cooperative traits could lead to improved fertilization success of sperm associated with a group, by enabling faster migration towards the site of fertilization^29^, and could be advantageous in sperm competition.

However, vital metrics of fertilization ability, including the DNA and membrane integrity, of sperm involved in cooperation remain elusive and are required to fully resolve how cooperative behavior is enabled and linked to fertilization success. Whether cooperation involves sperm with high or low DNA integrity can have a significant impact not only on fertilization success, but on the membrane integrity as well^35^, including its support of specific—and still unknown—surface entities that may mediate the attachment and cooperative behavior among sperm.

In humans, sperm cooperation is not expected—particularly given the rich literature on human sperm motion in the fields of hydrodynamics^36^, reproductive biology^8^, and clinical fertility treatment^37^. Human sperm have been shown to swim in a coordinated manner due to hydrodynamic interactions, as evidenced in experimental^15,38^ and computational analyses^39–41^, resembling that observed among sperm of sea urchins (*Strongylocentrotus droebachiensis* and *S. purpuratus*)^42^ and certain bacteria (*Bacillus subtilis*)^43^. In this mode, proximate sperm couples and synchronize their flagella via hydrodynamics, which results in collective motion^15^.

However, this method of collective swimming, based on flagellar synchronization, arises from proximity and hydrodynamics alone and thus is not cooperative behavior as exemplified in other species^24–30^. Cooperative swimming requires sperm to physically attach, followed by migration as a group with unbound flagella. Attachment is driven by morphological or membrane components, rather than by the hydrodynamics of the migrating medium. The progressive motion of the cooperative group with unbound flagella, regardless of synchrony, further distinguishes cooperative swimming from hydrodynamics-based collective swimming, which depends entirely on flagellar coupling and synchronization^15,38–41^.

Human sperm cooperation has also been refuted^44,45^. One report noted that while sperm aggregation can occur in intra- and inter-ejaculate samples, it is a result of incomplete liquefaction or sperm agglutination^44^, which arise due to an immunological response in the male reproductive tract and may cause infertility^44,46^. The study found an absence of cooperation or any selective interactions among human sperm. Another report suggested that the morphology of the human sperm head prevents cooperative swimming behavior^45^. Human sperm have a simple oval-shaped head that lacks distinct morphological features, such as a hook^28,31^, which could enable physical attachment. However, whether the membrane of human sperm, which is known to have distinct surface binding properties^47–51^, can mediate sperm attachment and cooperative behavior remains unproven.

We hypothesized that human sperm could benefit from cooperation in the highly viscous portions of the female reproductive tract^52^. At non-ovulatory times, the secretions of the female tract are highly viscous (~200 cP^19,53^). This viscous medium is prevalent in the early portions of the tract (i.e., vagina and cervix) and serves to block the tract from foreign organisms^52,54^. Upon insemination, an interface is formed between the seminal fluid (typically low viscosity, <10 cP^55^) and the high-viscosity medium. Under these conditions, sperm must migrate from the seminal fluid and transit the high viscosity medium to reach the site of fertilization near the oviduct and standby for ovulation (the period of 24–48 h where the ovum is ready for fertilization)^56,57^. At ovulation, the secretions within the female tract increase, by upwards of two- to three-fold^19^, and viscosity decreases, to as low as 2 cP in the oviduct^19^, to promote passage of sperm to the ovum and sperm-ovum engagement^52,58,59^. An ability to overcome the high-viscosity medium presented earlier in the cycle would improve the chances of fertilization. However, these viscosity contrasts in vivo are not replicated in standard in vitro methods in clinical or research settings that, instead, suspend sperm in a homogenous viscous medium for analyses.

Here, we report that human sperm can cooperate. We replicate the viscosity contrasts in the female reproductive tract^52^ using an in vitro microfluidic platform and find that individual sperm cooperate by attaching at the head region to migrate as a group upon transit into and through a high-viscosity medium from low-viscosity seminal fluid. Sperm groups benefit from increased swimming velocity, which exceeds that of individual sperm. We find that sperm associated with a group have significantly higher DNA integrity and membrane decapacitation factor levels than individual sperm, with the high presence of decapacitation factors mediating sperm attachment to form the group. The prevalence of cooperative behavior decreases due to capacitation or a reduction in the local viscosity. We examine the effect of inter-ejaculate conditions, revealing that related sperm have a high tendency to cooperate and form groups, with such groups exhibiting greater swimming velocity than groups consisting of unrelated sperm.

## Results and discussion

### Human sperm cooperation is viscosity driven

We devised an in vitro setup that interfaced low-viscosity seminal fluid with a higher-viscosity medium (15, 40, 65, or 100 cP), mimicking the viscosity interface that is present within the female tract at the onset of insemination (Supplementary Fig. 1b). We applied here microfluidic methods, established previously to mimic structures within the female tract for sperm analysis and selection^60–63^, to replicate the viscosity contrasts in the female tract. A laminar interface between the low-viscosity seminal fluid and the higher-viscosity medium was first established. The interface remained relatively intact with minimal diffusion and stagnant flow conditions within the 20 min experiment duration, while additional characterizations indicated that the interface could be maintained for up to 1.5 h (Supplementary Fig. 2).

**Fig. 1:**
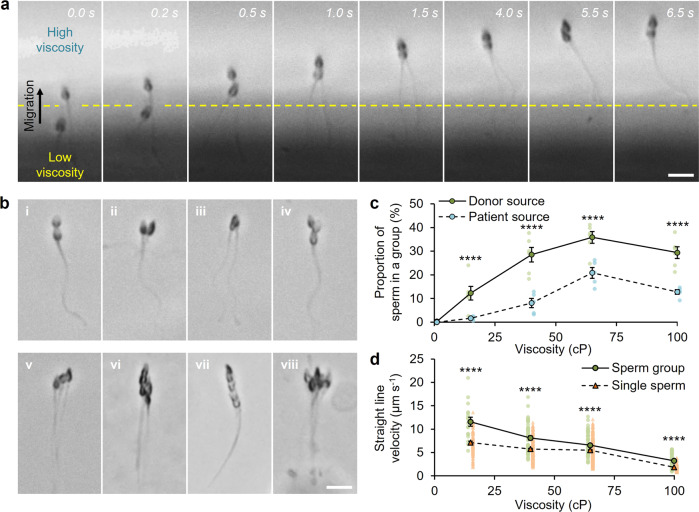
A viscous transition prompts cooperation among human sperm. **a** Formation of sperm groups. Individual sperm attach upon migration from low-viscosity seminal fluid into a higher-viscosity medium (15, 40, 65, or 100 cP), and form a group in 6.5 s. The average time for complete formation requires 7.1 s (±0.7 s, *n* = 3 independent replicates, *N* = 11 total sperm groups). Scale bar: 10 μm. **b** Sperm attachment occurs at the sperm head region (i–iii), and sperm groups feature various configurations and sizes (iv–viii). Scale bar: 10 μm. **c** Proportion of sperm belonging to a group in relation to the viscosity of the medium that sperm migrate through, of donor sources (*n* = 6 independent replicates, *N* = 2320–3208 total sperm per data point) and patient sources (*n* = 5 independent replicates, *N* = 6314–10,077 total sperm per data point). **d** Swimming velocity of sperm groups in comparison to single sperm as a function of the viscosity of the medium that sperm migrate through (*n* = 5 independent replicates, *N* = 17–69 total sperm groups per data point for “Sperm group” and 71–350 total individual sperm per data point for “Single sperm”; independent two-tailed Student’s *t*-test). Data were mean ± s.e.m. ****P < 0.0001.

Both donor (cryogenically frozen and thawed) and clinical patient samples (freshly ejaculated) were used. We found that as motile sperm from the seminal fluid of both donor and patient sources encounter the higher-viscosity medium (>15 cP), they slow and can attach to progress through the medium as a group (Fig. 1a). Sperm exhibit a planar waveform with reduced yaw (lateral movement) and rolling rate^15,53^, and approach at a 35–40° angle, in a manner that is one sperm slightly ahead of the other (Supplementary Fig. 3 and Supplementary Movie 1). The trailing sperm head attaches near the bottom of the leading sperm head, mainly in the midpiece region. Complete and stable formation of the group, whereby progressive swimming is achieved without detachment of sperm from the group, required 6.5 s in the case shown in Fig. 1a, and on average 7.1 s (±0.7 s, *n* = 3 independent replicates, *N* = 11 total sperm groups). The resulting groups were typically in a staggered configuration, with leading and trailing sperm, however also featured various other configurations and geometries (Fig. 1b and Supplementary Movies 2–4). Attachment did not prevent rolling entirely—the sperm group exhibited reduced rolling, while the flagella of sperm in a group were unbound and tended to beat either in asynchrony or synchrony as the group progressed. The tendency of sperm to cooperate increased at higher viscosities to a maximum of 65 cP. The proportion of sperm belonging to a group reached a peak of 36% (±3%, *n* = 6 independent replicates, *N* = 2872 total sperm) and 21% (±2%, *n* = 5 independent replicates, *N* = 1864 total sperm) for donor and patient sources, respectively, at 65 cP within a 20 min testing period (Fig. 1c). Sperm were observed to remain attached in the group in the high-viscosity medium (>15 cP) within the 20 min testing period.

The findings indicate that cooperative behavior occurs among human sperm from both donor and patient sources, is prompted by a low-to-high-viscosity transition, and increases in prevalence at higher viscosities. The process of group formation is enabled by: (1) sperm motion upon transition into the high-viscosity medium, in which the increased fluid drag promotes planar motion with reduced yaw and rolling rate, thereby increasing the longitudinal alignment for sperm to approach and attach; and (2) a physical attachment at the membrane surface.

The membrane characteristics may differ between cryopreserved and fresh sperm samples^64,65^. Quantification of group formation among sperm from both donor (frozen) and patient (fresh) samples revealed the occurrence of attachment for both sources despite the potential membrane differences. We note that the proportion of sperm in a group was significantly different (*P* < 0.0001) across the tested viscosities between donor and patient sources, with sperm from the donor sources exhibiting a higher tendency to be involved in a group than sperm from the patient sources, potentially due to the membrane differences or the lower motility level of post-thaw sperm. The post-thaw donor sources featured an average of 30.5% total motile sperm and a concentration of 20.2 million sperm per ml, while the fresh patient sources featured an average of 51.8% total motile sperm and a concentration of 77.4 million sperm per ml. The reduced amounts of motile and total sperm in the donor sources contribute to a lower sperm count for analysis (*N* = 2320–3208 total sperm analyzed per data point in Fig. 1c) in comparison to that of the patient sources (*N* = 6314–10,077 total sperm analyzed per data point in Fig. 1c). It is noteworthy that the lower sperm count in the post-thaw donor samples facilitated a more pronounced grouping behavior by allowing motile sperm to readily approach one another and attach. In contrast, a high sperm count, as in the fresh samples, resulted in overcrowding and increased the likelihood of sperm collision and deflection rather than the required approach and attachment behavior.

The cooperative swimming behavior of a human sperm group was the same for both donor and patient sources, and resembled that of the few other animal species that are known to exhibit cooperation^24–30^— sperm physically attach and progressively swim as a group with unbound flagella, irrespective of beat synchrony. This behavior is distinct from the more common case of collective swimming of sperm based on hydrodynamic interactions, which relies on flagellar synchronization for motion^15,38–41^.

The progressive swimming velocity (straight-line velocity) of the sperm groups was examined and compared to that of individual sperm not in a group. Sperm groups exhibited a higher swimming velocity than single sperm, by an average of 51% (±13%) for all tested viscosities (Fig. 1d). The cooperative effort of sperm in a group would seem to benefit the entire group, as denoted by the higher swimming velocity of the sperm group.

The increase in swimming velocity could be attributed to a reduction in the drag force, based on the resistive movement and lateral forces, experienced by the sperm group. Similarly, hydrodynamic coupling of sperm reduces yaw motion in the sperm body, enables a bi-directional flagellar waveform, and increases swimming velocity^15,66^. Compact attachment at the sperm head regions could likewise restrict the lateral movement of sperm heads within a group^67^. These factors could increase the longitudinal alignment, persistence, and propulsion of the sperm group to promote a more ATP-efficient swimming behavior^66^ that improves swimming efficiency and velocity. It is noteworthy that the grouping behavior also provided a competitive advantage for sperm exhibiting only weak twitching motility after forming a group. For instance, the trailing sperm on the left side of the group in Supplementary Movie 4 was relatively immotile with weak twitching motility (slow head rotation) but was still moving progressively with the group.

By swimming as a group, sperm can migrate more efficiently, consuming less ATP on an individual basis^38,68^. Thus, sperm that cooperate may possess increased fertilization ability. Faster and more ATP-efficient transit as a group would allow sperm to overcome highly viscous conditions, migrate more efficiently to the site of fertilization, and thereby outcompete individual swimmers. Sperm that conserve more ATP in transport, by associating with a group, could have a greater capability to maintain motility^68,69^ and undergo capacitation^70^ to engage with the ovum, once available.

The sperm group swimming velocity as a function of the group size was also examined. While group formation generally led to an increase in swimming velocity, the extent of the benefit differed according to the group size. Smaller groups consisting of two to three sperm exhibited a velocity that was 27% higher than larger groups with four to seven sperm (Supplementary Fig. 4). The findings suggest that group size and the resulting change in the group geometry and coordination can affect the swimming velocity. Smaller groups can possess a geometry with restricted and reduced lateral movement, thereby facilitating a linear and longitudinal swimming trajectory^71^. As a group becomes larger, it can inherit a wider set of configurations and a more unrestricted geometry, which may lead to opposing forces among sperm in the group^71^, deterring the motion of both the individual sperm involved and the entire group. The results also indicate the prevalence of an optimal group size of two to three sperm, which were frequently formed (92% of the total sperm groups analyzed), leading to the highest increase in swimming velocity.

We note that the viscous medium used in the in vitro setup was based on polyvinylpyrrolidone (PVP). PVP medium is a clinical standard synthetic secretory fluid used in in vitro investigations of sperm motion in viscous conditions as well as in assisted reproduction^72,73^. To validate that sperm group formation was driven by viscosity and not by the PVP chemistry, we performed additional experiments using a 1% methylcellulose (MC)^74^ medium with a nominal viscosity of 100 cP. Sperm exhibited similar behavior as in PVP and formed groups upon transition from the seminal fluid into the MC medium (Supplementary Movies 5–7). However, the prevalence of sperm groups was considerably lower in MC when compared to PVP. Several factors could contribute to this outcome, including the different rheological properties of the media and the chemical composition of the media that could potentially change the surface properties (e.g., electrostatic charge) of the sperm to mediate the grouping behavior. MC has shear-thinning properties^75^, which indicates that the proximate viscosity surrounding the sperm is lower due to motion. The reduced viscosity increases yaw and lateral forces, and decreases longitudinal alignment, in the sperm motion that can potentially destabilize or prevent attachment. In contrast, PVP has constant viscosity independent of shear strain^76^. Thus, the sperm experiences a constant viscosity, and at high viscosities (>65 cP), the sperm motion is suppressed and aligned longitudinally, and group formation is facilitated.

### Sperm with high DNA integrity and membrane decapacitation factors form cooperative groups

**Fig. 2:**
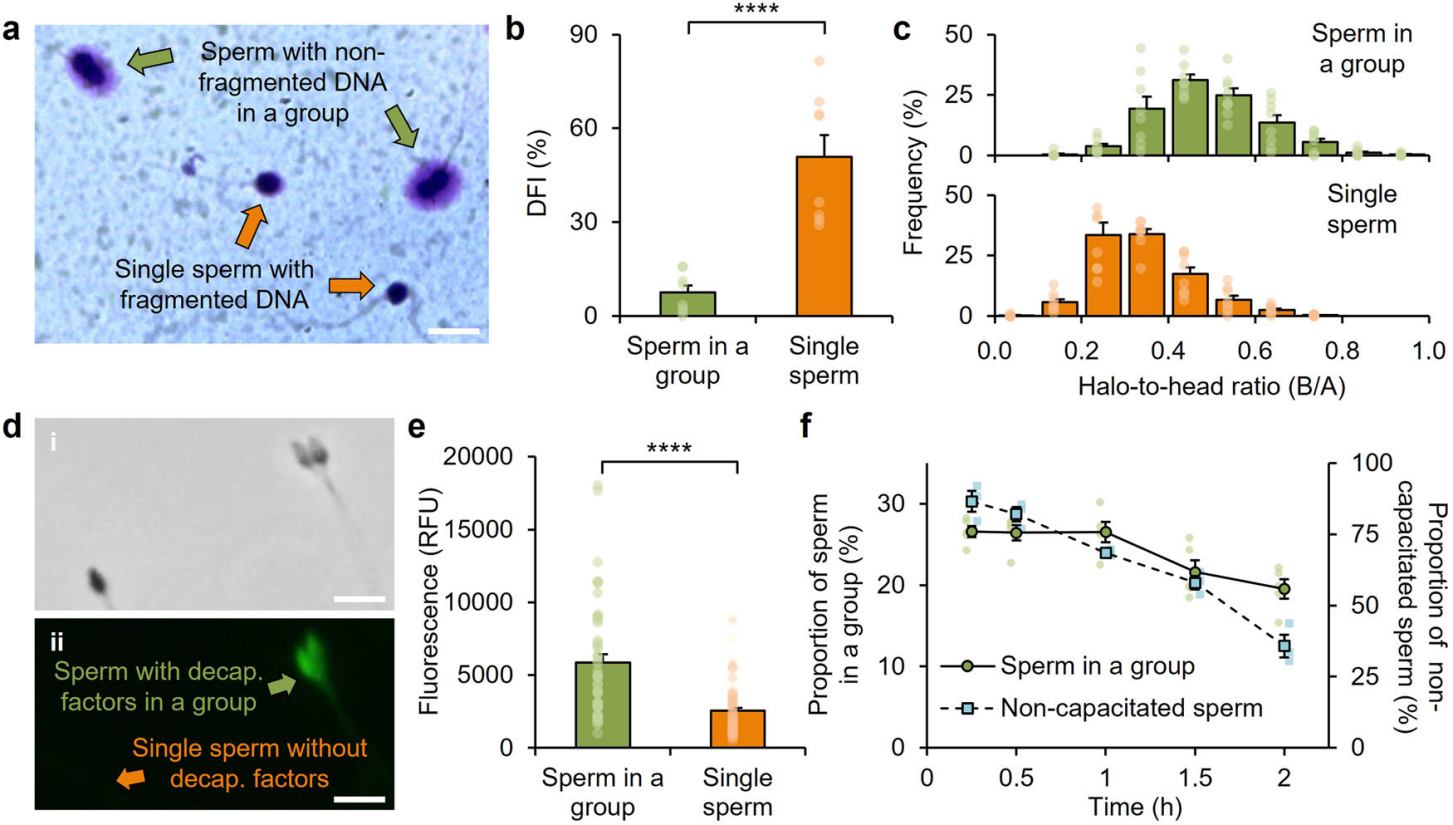
Cooperation involves sperm with high DNA integrity and membrane decapacitation factors. **a** Sperm groups feature sperm with non-fragmented DNA, while single sperm possess fragmented DNA. Scale bar: 10 μm. **b** Proportion of sperm with fragmented DNA (DNA fragmentation index, DFI) of sperm belonging to a group and single sperm (*n* = 8 independent replicates, *N* = 821 total sperm for “Sperm in a group” and 860 total sperm for “Single sperm”; independent two-tailed Student’s *t*-test). **c** Distribution of halo-to-head ratios (B/A values) of sperm in a group and single sperm (*n* = 8 independent replicates, *N* = 821 total sperm for “Sperm in a group” and 860 total sperm for “Single sperm”). **d** Representative grayscale (i) and fluorescence (ii) images of highly stained sperm with decapacitation factors belonging to a group and a minimally stained single sperm without decapacitation factors. Scale bars: 10 μm. **e** Fluorescence intensity of sperm belonging to a group in comparison to single sperm (*n* = 3 independent replicates, *N* = 48 total sperm for “Sperm in a group” and 79 total sperm for “Single sperm”; independent two-tailed Student’s *t*-test). **f** Proportion of sperm belonging to a group (*n* = 5 independent replicates, *N* = 1010–3021 total sperm per data point) and non-capacitated sperm (*n* = 3 independent replicates, *N* = 146–296 total sperm per data point) over a period of 2 h. Data were mean ± s.e.m. ****P < 0.0001.

To examine sperm DNA integrity, we configured an in vitro setup that supported sperm group formation, fixation, and DNA assessment via the sperm chromatin dispersion (SCD) assay^77,78^. For group formation, seminal fluid was interfaced with a 65 cP medium—the viscosity that enabled peak tendency for cooperative behavior (Fig. 1c). SCD quantifies the fragmentation level in sperm DNA, as represented by the ratio between the produced “halo” of dispersed DNA (termed dimension “B”) and the sperm head (termed dimension “A”)^77,78^, or the halo-to-head ratio (B/A value). Sperm with non-fragmented DNA were prevalent among those belonging to a group, while individual sperm frequently exhibited fragmented DNA (Fig. 2). The proportion of sperm with fragmented DNA, or percent DNA fragmentation index (DFI), of sperm in a group was on average 7% (*n* = 8 independent replicates, *N* = 821 total sperm), which was markedly lower than that of single sperm with a DFI of 51% (*n* = 8 independent replicates, *N* = 860 total sperm; *P* < 0.0001) (Fig. 2b). The distribution of B/A values (normal distributions as verified using the Kolmogorov–Smirnov Test, Supplementary Fig. 5) revealed that 45% of sperm in a group possessed non-fragmented DNA with high integrity (B/A > 0.5), as opposed to individual sperm that only featured 9% (Fig. 2c).

These findings indicate that sperm with high DNA integrity are predominantly involved in cooperative behavior, and that this behavior could be governed by the plasma membrane characteristics associated with high sperm DNA integrity. Sperm with high DNA integrity have high membrane integrity^35^ and are known to produce low levels of reactive oxygen species (ROS), which are required for capacitation^79–81^—a physiological change involving a reorganization of decapacitation factors on the membrane that prepares sperm for fertilization at ovulation^82,83^. In contrast, sperm with low DNA integrity signifies an excess production of ROS, which causes damage to the DNA and the membrane, obscuring capacitation and support for the necessary decapacitation factors^79–81^. We reasoned that decapacitation factors on the membrane could be vital to the cooperative behavior among sperm. Decapacitation factors maintain sperm stability and survival during transit^83^, and possess binding properties—for instance, enabling sperm to bind to the oviduct epithelium and form a sperm reservoir within the oviduct^47,84^—which could also potentially mediate attachment among sperm to form a cooperative group.

We assessed the role of decapacitation factors in cooperative behavior by first evaluating the level of decapacitation factors of sperm associated with a group in comparison to individual sperm. We quantified the level of cholesterol as a proxy for the overall level of decapacitation factors on the sperm membrane by fluorescently labeling sperm with BODIPY-cholesterol^85^. Sperm with high signal (5845 ± 575 RFU, *n* = 3 independent replicates, *N* = 48 total sperm), indicating a high level of decapacitation factors, were typically associated with a group (Fig. 2d, e). In contrast, individual sperm exhibited low signal (2555 ± 199 RFU, *n* = 3 independent replicates, *N* = 79 total sperm; *P* < 0.0001), indicating a low level or absence of decapacitation factors.

Next, we examined how sperm capacitation, including the removal of decapacitation factors, over a period of 2 h would affect the tendency of sperm to form groups. Capacitation was induced by prolonged incubation in the medium, which was pre-supplemented with capacitation-promoting factors, including albumin and bicarbonate. We assessed only motile sperm groups and individual sperm at each testing time point to avoid potential inaccuracies in the analyses due to sperm death over time. The proportion of sperm belonging to a group was negatively associated with the testing time, decreasing by 27% from 0.25 to 2 h (Fig. 2f). The proportion of total sperm with decapacitation factors, or non-capacitated sperm, presently followed a similar negative trend, decreasing by 59% from 0.25 to 2 h (Fig. 2f).

These findings suggest that decapacitation factors, including but not limited to cholesterol, on the sperm membrane mediate the ability of sperm to attach and form cooperative groups, which is further evidenced by the loss of the cooperative ability due to capacitation and the removal of decapacitation factors.

Taken together, the results reveal that sperm with high DNA integrity can have high membrane integrity that supports decapacitation factors, which enable the ability to form cooperative groups to gain the competitive advantage of enhanced swimming velocity. The fertilization progression and timeline can now include a cooperative approach. At the start of the fertilization journey and during non-ovulatory periods, sperm with high DNA integrity and decapacitation factors engage in cooperation that enables faster migration through the high-viscosity secretions leading to the oviduct. Upon reaching the oviduct, and at the onset of ovulation (characterized by an increase in capacitation-promoting factors^83,86^ and a decrease in secretion viscosity^52,58,59^), sperm undergo capacitation, cease cooperation, and swim individually in competition through a low-viscosity medium to engage with the ovum.

### Sperm cease cooperative behavior upon entering a lower-viscosity medium

Ovulation involves an increase of secretions with capacitation-promoting factors that induce sperm capacitation^83,86^—a potential trigger to cease cooperative behavior—and a decrease in secretion viscosity^52,58,59^. We investigated whether the decrease in viscosity associated with ovulation could additionally prompt sperm to cease cooperative behavior.

**Fig. 3:**
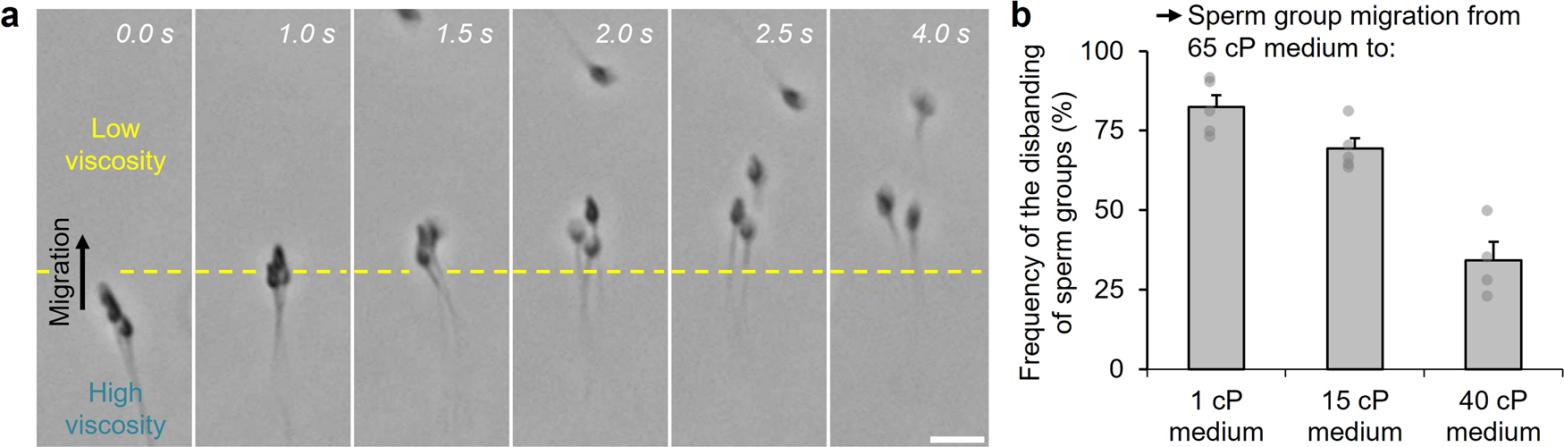
Sperm groups disband due to a reduction in the local viscosity. **a** Sperm group disbands upon migration from a high-viscosity medium into a low-viscosity medium, requiring 4.0 s for all sperm to detach. The average time for detachment of all sperm requires 3.6 s (±0.4 s, *n* = 3 independent replicates, *N* = 13 total sperm groups). Scale bar: 10 μm. **b** Frequency at which sperm group disbanding occurs when migrating from a 65 cP medium to a 1, 15, or 40 cP medium (*n* = 5 independent replicates, *N* = 76 total sperm groups for “1 cP medium”, 87 total sperm groups for “15 cP medium”, and 83 total sperm groups for “40 cP medium”). Data were mean ± s.e.m.

We sequentially interfaced seminal fluid with a higher-viscosity medium (65 cP) and then a lower-viscosity medium (1, 15, or 40 cP), mimicking the high-to-low-viscosity change within the female tract at ovulation (Supplementary Fig. 1c). Sperm from the seminal fluid initially formed groups upon migration through the 65 cP medium. Upon further migration to the lower-viscosity medium, sperm groups tended to disband, whereby sperm detached from their respective groups and swam individually (Fig. 3a). Complete disbanding of the group, signified by the detachment of all sperm from the group, required 4.0 s in the case shown in Fig. 3a, and on average 3.6 s (±0.4 s, *n* = 3 independent replicates, *N* = 13 total sperm groups). The prevalence of disbanding increased as the viscosity of the medium that sperm groups migrated into decreased, reaching 82% (±4%, *n* = 5 independent replicates, *N* = 76 total sperm groups) for a medium with a viscosity of 1 cP (Fig. 3b).

These findings indicate that viscosity enables and disables cooperative behavior. The high-viscosity transition drives sperm that are in the initial non-capacitated state, with decapacitation factors, to cooperate and attach. Subsequently, sperm can cease to cooperate over a longer period due to capacitation (and loss of decapacitation factors) or more immediately in response to a reduction in the local viscosity—the latter prompting sperm detachment regardless of capacitation status.

Sperm detachment could be triggered at lower viscosities due to an increase in yaw, rolling, and lateral movement of individual sperm within the group. Yaw discourages the longitudinal alignment of the sperm body and facilitates lateral movement of the sperm head region^15,54,66^ that could weaken the attachment established by the decapacitation factors, causing sperm to detach. The amount of yaw in sperm motion increases, which in turn could cause a higher tendency for sperm to separate from their groups, as viscosity and the associated fluid drag forces decrease^15,54,66^. In the case where a sperm group remained intact following migration into a lower-viscosity medium, the group exhibited decreased planarity and increased rolling, including 3D movement and rotation, and yaw (Supplementary Movie 8). Sperm involved in the group displayed near identical rolling frequencies, with the sperm heads rotating about the point of contact where the attachment was achieved. The disbanding of the sperm group based on sperm capacitation and a reduction in the local viscosity could signify a transition from cooperation to individual competition among sperm in the group at ovulation.

Additional characterization was performed to validate that sperm detachment was driven by a viscosity reduction, rather than by a decrease in PVP concentration. An interface was established between viscous media based on PVP and MC of ~65 cP nominal viscosity. Sperm group migration between PVP and MC led to no disbanding (Supplementary Movie 9). In the case of migration from 65 cP PVP to 50 cP MC, the sperm in the group exhibited significant yaw, particularly in the head region, causing the group to disband (Supplementary Movie 10). While the chemical composition of the medium might also contribute, our findings suggest that sperm detachment is mainly mediated by a reduction in the local viscosity.

We also assessed sperm group motion upon migration back into the low-viscosity seminal fluid. Sperm groups tended to disband upon encountering the seminal fluid (Supplementary Movies 11, 12), reflecting the disbanding behavior as observed in low-viscosity media (Fig. 3). The findings indicate that cooperative sperm groups are not present in the original seminal fluid, and further support the notions that cooperative swimming behavior is driven by a high-viscosity transition and ceases in low-viscosity conditions.

### Inter-ejaculate conditions promote cooperation among related sperm

We also investigated whether human sperm cooperate based on relatedness. Promiscuity raises the potential of inter-ejaculate sperm competition between males for copulation or mating privileges^1–3,10^. To assess cooperation among related sperm, we established inter-ejaculate conditions, prepared with a seminal fluid mixture containing sperm from two male sources. Sperm from one sample were labeled with a fluorescent stain (SYBR-14 Live Cell Stain) and sperm from the other sample were left unstained (Supplementary Fig. 6). For the control, we prepared a seminal fluid mixture containing sperm from only one male sample, with sperm from one part of the sample stained and the other part unstained.

**Fig. 4:**
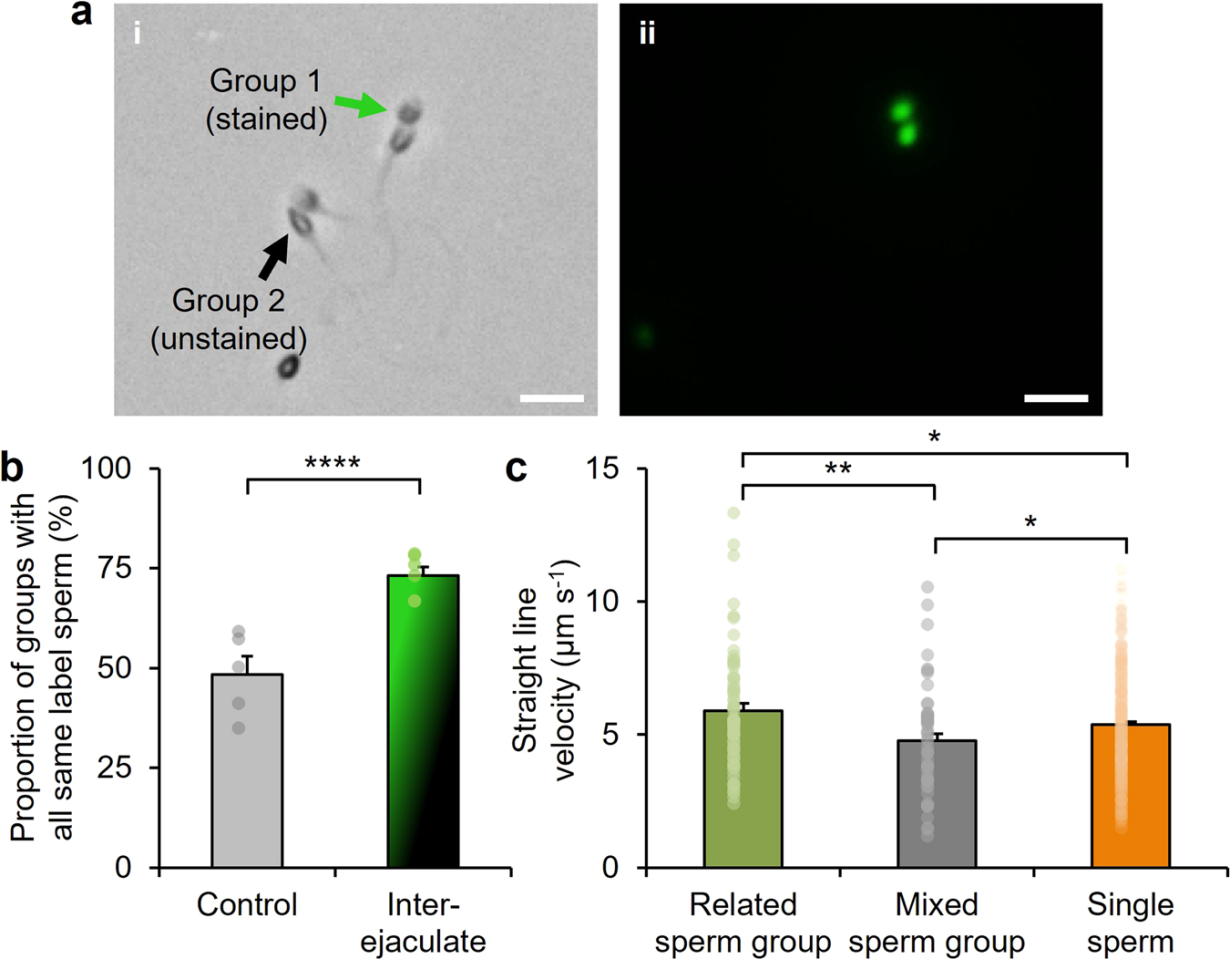
Sperm cooperate based on relatedness. **a** Representative grayscale (i) and fluorescence (ii) images of sperm groups formed based on an inter-ejaculate mixture upon migration into a 65 cP medium. To distinguish sperm from the different samples, sperm from one sample were labeled with a fluorescent stain (“Group 1”) and sperm from another sample were unstained (“Group 2”). Scale bars: 10 μm. **b** Proportion of sperm groups with all the same-label sperm (for “Control”: *n* = 5 independent replicates, *N* = 112 total sperm groups; for “Inter-ejaculate”: *n* = 6 independent replicates, *N* = 169 total sperm groups; independent two-tailed Student’s *t*-test). For the control, the same male sample was split, whereby sperm from one part were labeled with a fluorescent stain and the other part were kept unstained, and subsequently re-combined for testing. **c** Swimming velocity of sperm groups consisting of related sperm, sperm groups consisting of unrelated sperm, and single sperm not associated with a group (*n* = 5 independent replicates, *N* = 68 total sperm groups for “Related sperm group”, 55 total sperm groups for “Mixed sperm group”, and 330 total individual sperm for “Single sperm”; independent two-tailed Student’s *t*-test). Data were mean ± s.e.m. ****P < 0.0001; **P < 0.01; *P < 0.05.

We examined the composition of sperm groups, which revealed that related sperm had a higher tendency to form groups than unrelated sperm in inter-ejaculate conditions. Of the total number of sperm groups, 73% (±2%, *n* = 6 independent replicates, *N* = 169 total sperm groups; *P* < 0.0001) were formed entirely of sperm that were either all stained or all unstained (Fig. 4a, b). This large degree of similarity among sperm that form a group is remarkable and in stark contrast to the control and a statistically predicted random association model, which suggest that without such preference for relatedness in inter-ejaculate conditions, the degree of similarity would be 48% (±5%, *n* = 5 independent replicates, *N* = 112 total sperm groups) and 43% (±3%) (Supplementary Fig. 7), respectively.

The prevalence of cooperation among related human sperm in inter-ejaculate conditions suggests that sperm attachment is preferential and selective based on relatedness. Such attachment could be a consequence of homophilic binding from genetic similarities among related sperm^87–89^. Cells featuring the same genes for homophilic binding can selectively distinguish and bind with each other to gain a cooperative benefit^87^. This binding mechanism has been observed in microorganisms such as amebas^90^, yeast^91^, and bacteria^92^, and has been suggested to contribute to preferential cooperation among related sperm of deer mice (*Peromyscus maniculatus*)^33^. The attachment among human sperm in inter-ejaculate conditions may be mediated by surface mechanisms, including decapacitation factors (as a general attachment mechanism) and homophilic molecules (as a genetic-based attachment mechanism^87–89^). It is noteworthy that for sperm from the same source (Fig. 2d, e), the prevalence of decapacitation factors, including cholesterol, among sperm in a group that were all related obscured the observation of preferential attachment.

Groups consisting of only related, same-label sperm (related sperm group) exhibited an average swimming velocity that was greater by 24% over groups consisting of unrelated, different-label sperm (mixed sperm group) in a 65 cP medium (Fig. 4c). Compared to sperm that proceeded alone, the average swimming velocity of the related group was higher by 10%, while the mixed group was lower by 11%. It is surprising that relatedness so markedly influenced the swimming velocity—related sperm benefited from swimming as a group, while unrelated sperm were slowed by their involvement in a group. These observations suggest that there could be an intercellular mechanism specific among related or unrelated sperm that may regulate the motion of sperm in a group and thereby influence the swimming effectiveness of the collective.

This ability to cooperate based on relatedness to improve fertilization ability and outcompete rival sperm resembles the ‘helper’ and ‘inhibitor’ functions of sperm in other species such as worms^93^ and snails^94^. While it has been suggested that these functions are unlikely to be exhibited by human sperm^44^, our results provide an intriguing case for further discussion and investigations.

## Conclusion

We found that human sperm cooperate, forming motile swimming groups when prompted by conditions that mimic the viscosity contrasts in the female tract. Individual sperm attached and continued migration as a group upon transit into and through a high-viscosity medium from low-viscosity seminal fluid. Sperm groups gained a cooperative benefit of enhanced swimming velocity that was over 50% higher than individual sperm. Sperm associated with a group possessed high DNA integrity (7% DFI), as opposed to individual sperm that exhibited low DNA integrity (>50% DFI). The ability to attach and cooperate was dependent on the presence of decapacitation factors on the sperm membrane. Cooperative behavior ceased to occur over a longer period due to capacitation or immediately due to a reduction in the local viscosity. Under inter-ejaculate conditions, related sperm displayed a high tendency to form groups, whereby 73% of sperm groups featured only sperm from the same male, and benefit from higher swimming velocity. In contrast, unrelated sperm were, on average, slowed by their association with a group.

These findings suggest a cooperation-based migration strategy for human sperm in the fertilization progression and timeline. Sperm with high DNA integrity possessing decapacitation factors can cooperate to migrate more efficiently through the highly viscous conditions of the female tract to outcompete rival sperm in reaching the site of fertilization. Thus, cooperation can allow for sperm of high fertilization ability to engage with the ovum at ovulation.

These findings also provide insight into how cooperation could be used as a mechanism for the selection of sperm with high DNA integrity to improve assisted reproduction processes and success^95–98^. Our study uncovers cooperation among human sperm and highlights its potential implications for human reproduction.

## Methods

### Sperm preparation

Donor semen samples were obtained from ReproMed Ltd (Toronto, Canada) in a cryogenically frozen state and stored in a freezer at −80 °C until use. The samples were thawed at 37 °C for 20 min prior to each experiment. The post-thaw donor sources featured an average of 30.5% total motile sperm and 20.2 million sperm concentration. Patient semen samples were obtained from the Hannam Fertility Clinic (Toronto, Canada). The samples were fresh and immediately used for testing. The fresh patient sources featured an average of 51.8% total motile sperm and 77.4 million sperm concentration. Donors and patients provided informed consent for research purposes in accordance with the regulations of the Assisted Human Reproduction Act (approval number: 37629). The University of Toronto Biosafety Office approved the experimental use of donor and patient samples.

### Medium preparation

The primary medium used for testing was prepared by dissolving polyvinylpyrrolidone (PVP) powder (Sigma Aldrich, Canada) in PureSperm medium (Nidacon, Canada). Different concentrations of PVP were used to vary the viscosity of the medium, including 3, 5, 7, and 10% PVP, which correspond to 15, 40, 65, and 100 cP, respectively, as quantified through rheological assessments. Methylcellulose medium was prepared by dissolving methylcellulose (MC) powder (Sigma Aldrich, Canada) in PureSperm medium (Nidacon, Canada), with concentrations ranging between 0.5–1%.

### In vitro microfluidic platform

We developed a microfluidic platform consisting of a straight microchannel (5 mm length, 100 μm height, and 1 mm width) and an open inlet region (Supplementary Fig. 1a). The design was patterned into a biocompatible film (PCR opto-adhesive film; MedStore, Canada) using a cutting plotter (Silhouette Studio 3; Silhouette Inc., Canada), which was then applied onto a microscope glass slide.

### Viscous interface characterization

We examined the persistence of the laminar interface established between media of varying viscosities. The microchannel was first filled with a higher-viscosity medium (15, 65, or 100 cP) by capillary action. The inlet was then loaded with a stock medium of low viscosity (1 cP), which was pre-mixed with fluorescent microbeads (1:100 dilution) that allowed accurate tracking of the fluid interface. Each setup (i.e., the interface between 1 cP to 15, 65, or 100 cP) was examined to determine the distance that the interface shifted (via diffusion) from the channel entrance within a 1.5 h testing time (Supplementary Fig. 2).

We computed the diffusion coefficient (D) based on Brownian motion by assessing the mean square displacement of the microbeads over time using the following relationship:

1 $${x}^{2}=2{Dt}$$

where

*x* is the distance that the microbeads have moved

*t* is the duration, and

*D* is the diffusion coefficient.

### Sperm group formation assay

We established an in vitro setup that interfaced seminal fluid and a higher-viscosity medium (Supplementary Fig. 1b). The microchannel was first filled with a higher-viscosity medium (15, 40, 65, or 100 cP) by capillary action. The inlet was then loaded with raw semen (20 μL) that interfaced with the medium at the entrance of the channel. This setup was incubated at 37 °C for 20 min and subsequently analyzed using an EVOS FL Auto Imaging Microscope (Thermo Fisher Scientific, Canada) at 20× magnification. Sperm groups and individual sperm were located, and short videos of their swimming trajectories were recorded. The proportion of sperm in a group was computed as the number of sperm belonging to a group out of the total between sperm belonging to a group and individual sperm. The straight-line swimming velocity of sperm groups and individual sperm were calculated by evaluating their straight swimming distances achieved in 5 s, obtained through recordings. These recordings were converted to image sequences and then imported to ImageJ for velocity analyses.

### Sperm DNA integrity assay

We configured an in vitro setup that enabled sperm group formation, with subsequent fixation and DNA assessment. A 65 cP medium (40 μL) was loaded onto an agarose-coated microscope slide. A droplet of raw semen (5 μL) was carefully suspended in the 65 cP medium, without mixing to ensure an interface between seminal fluid and the 65 cP medium. The setup was incubated at 37 °C for 20 min to allow sperm groups to form. The setup was then quickly dried via storage in a vacuum chamber (~10 min evaporation time), leaving behind a thin film with immobilized sperm groups on the slide. For fixation, 1% low melt agarose solution (30 μL) was loaded onto the film and allowed to gelate. Sperm were embedded within the gel, which was firmly attached to the agarose coat on the slide.

We assessed sperm DNA integrity directly following fixation using the sperm chromatin dispersion (SCD) assay (SCD kit; Fertitech, Canada). The slide was introduced to an acid-denaturing solution (1 mL; 0.08 N HCl) for 30 min at room temperature, followed by a neutralizing and lysing solution (1 mL; 0.4 M Tris, 0.8 M DTT, 1% SDS, and 50 mM EDTA, pH 7.5) for 18–20 h at 4 °C. The slide was placed in distilled water for 5 min and then in 70, 90, and 100% ethanol for 2 min each, and dried in nature. For staining, Wright’s stain (300 μL) and Wright’s buffer (750 μL) were dispensed onto the slide. After 20 min, the slide was rinsed with distilled water and dried in nature. The slide was analyzed using the EVOS microscope at 20× magnification. Stained sperm, with various halo sizes, were located and images were taken. The images were imported into ImageJ to analyze and measure the size of the halo and sperm head for each sperm associated with a group and individual sperm. The halo-to-head ratios (B/A values) were then computed. The DNA fragmentation index (DFI) was computed as the number of sperm with fragmented DNA (B/A < 0.33) out of the total between the number of sperm with fragmented DNA and non-fragmented DNA (B/A > 0.33). The distribution of halo-to-head ratios was computed by organizing the B/A values into a histogram.

### Sperm decapacitation factors and capacitation assays

The stock solution of BODIPY-cholesterol (Sigma Aldrich, Canada) was prepared by reconstitution in DMSO to a concentration of 1 mM. The stock solution was stored in a freezer at −80 °C until use and thawed at 37 °C for 20 min prior to use. For sperm staining, BODIPY-cholesterol was added to raw semen (final concentration of 1 μM). The resulting mixture was incubated at 37 °C for 40 min.

For experiments assessing the level of decapacitation factors of sperm belonging to a group in comparison to individual sperm, we introduced the stained mixture into the in vitro platform and evaluated the fluorescence intensity of sperm associated with a group and individual sperm. The microchannel was filled with 65 cP medium by capillary action. The mixture (20 μL) was loaded at the inlet, interfacing with the 65 cP medium at the channel entrance. This setup was incubated at 37 °C for 20 min and subsequently examined using the EVOS microscope at 20× magnification. Motile sperm groups and individual sperm were located and imaged in fluorescence mode. The images were imported into ImageJ, which was used to assess the fluorescence intensities of sperm belonging to a group and individual sperm.

For experiments assessing the proportion of sperm belonging to a group over 2 h, we introduced raw semen (non-labeled sperm) into the in vitro platform and evaluated the number of sperm associated with a group over a period of 2 h. The microchannel was filled with 65 cP medium and the inlet was loaded with raw semen (20 μL). This setup was incubated at 37 °C for 2 h. During incubation, the setup was examined at specific time points (0.25, 0.5, 1, 1.5, and 2 h) using the EVOS microscope at 20× magnification. Only motile sperm groups and individual sperm were assessed. The proportion of sperm in a group was computed as the number of sperm belonging to a group out of the total between sperm belonging to a group and individual sperm.

For experiments assessing the proportion of total non-capacitated (highly stained) sperm present over 2 h, we introduced a stained sperm suspension, with seminal fluid removed, into a 65 cP medium and evaluated the number of highly stained sperm over a period of 2 h. Seminal fluid from stained raw semen (100 μL) was removed by centrifugation (300 × *g* for 5 min) and the pellet was resuspended in a 65 cP medium (250 μL). This suspension was incubated at 37 °C for 2 h. At specific time points (0.25, 0.5, 1, 1.5, and 2 h), an aliquot (10 μL) of the suspension was dispensed onto a glass slide and examined using the EVOS microscope at 20× magnification. Motile sperm were located and viewed in both grayscale and fluorescence modes. The proportion of highly stained sperm was computed as the number of sperm displaying a high fluorescence out of the total between sperm displaying a high fluorescence and sperm with minimal fluorescence.

### Sperm group disbanding assay

We established an in vitro setup that interfaced seminal fluid, a 65 cP medium, and a lower-viscosity medium (1, 15, or 40 cP) (Supplementary Fig. 1c). The microchannel was first filled with a lower-viscosity medium (1, 15, or 40 cP) by capillary action. The inlet was loaded with a 65 cP medium (40 μL) that interfaced with the lower-viscosity medium at the entrance of the channel. A droplet of raw semen (20 μL) was carefully suspended in the 65 cP medium, without mixing to ensure an interface between seminal fluid and the 65 cP medium. This setup was monitored for 20 min using the EVOS microscope at 20× magnification. We examined sperm migration between the seminal fluid and the 65 cP medium (sperm group formation), and between the 65 cP medium and the lower-viscosity medium (sperm group disbanding). The frequency whereby sperm group disbanding occurs was computed as the number of successful disbanding events out of the total between successful and unsuccessful disbanding events, upon sperm group migration into the lower-viscosity medium.

### Inter-ejaculate sperm group formation assay

Two different donor semen samples were chosen based on comparable live concentrations and total motile counts. To distinguish sperm from the different samples, we labeled sperm from one sample with an SYBR-14 live cell fluorescent stain (Sigma Aldrich, Canada) and kept sperm from the other sample unstained. For labeling, SYBR-14 (1 μL) was added to the sperm suspension (99 μL) and the mixture was incubated at 37 °C for 8 min.

To establish inter-ejaculate conditions, sperm from the two samples (100 μL for each sample) were combined and incubated at 37 °C for 20 min. For the control, the same sample was split, whereby sperm from one part were labeled with SYBR-14 and sperm from the other part were kept unstained, and combined. We incorporated inter-ejaculate conditions into the in vitro platform and evaluated the composition of sperm associated with a group. The microchannel was filled with 65 cP medium and the inlet was loaded with seminal fluid containing mixed-sample sperm (20 μL), or in the case of control, same-sample sperm (20 μL). This setup was incubated at 37 °C for 20 min and subsequently analyzed using the EVOS microscope at 20× magnification. Motile sperm groups were located and viewed in both phase contrast (grayscale image) and green fluorescence modes. The swimming trajectories of the sperm groups were recorded. The proportion of groups with all same-label sperm was computed as the number of sperm groups that were formed entirely of sperm that were either all stained or all unstained out of the total between sperm groups that consisted of all same-label sperm and different-label sperm.

We computed the swimming velocity (straight-line velocity) of sperm groups consisting of same-label sperm (related sperm group) and sperm groups consisting of different-label sperm (mixed sperm group) by evaluating their straight swimming distances achieved in 5 s from the recordings. These recordings were converted to image sequences and then imported to ImageJ for velocity analyses.

### Statistics and reproducibility

All data are mean ± s.e.m. The sample size and *n* values are indicated in the respective figure legends. All statistical analyses were performed in GraphPad Prism 7.0. To assess the significance between the groups of data, an independent two-tailed Student’s *t*-test was performed. Statistical significance was set as ****P < 0.0001; **P < 0.01; *P < 0.05.

### Reporting summary

Further information on research design is available in the Nature Portfolio Reporting Summary linked to this article.

## Supplementary information

Supplementary Information

Description of Additional Supplementary Data

Supplementary Data 1

Supplementary Movie 1

Supplementary Movie 2

Supplementary Movie 3

Supplementary Movie 4

Supplementary Movie 5

Supplementary Movie 6

Supplementary Movie 7

Supplementary Movie 8

Supplementary Movie 9

Supplementary Movie 10

Supplementary Movie 11

Supplementary Movie 12

Reporting Summary

## Data Availability

The main data supporting the results of this study are available within the paper and its Supplementary Information. The raw and analysed datasets generated during the study are provided in Supplementary Data 1.
