# Supplementary material for: Human sperm cooperate to transit highly viscous regions on the competitive pathway to fertilization
Source: Commun Biol. 2023 May 6;6:495. doi: 10.1038/s42003-023-04875-2 (PMC10164193; doi:10.1038/s42003-023-04875-2)
Supplement: Supplementary file 1 — Supplementary Information [file 42003_2023_4875_MOESM1_ESM.pdf]

Supplementary information for

Human sperm cooperate to transit highly viscous regions on the competitive pathway to fertilization

Sa Xiao, Jason Riordon, Alex Lagunov, Mahta Ghaffarzadeh, Thomas Hannam, Reza Nosrati and David Sinton

Correspondence to: sinton@mie.utoronto.ca

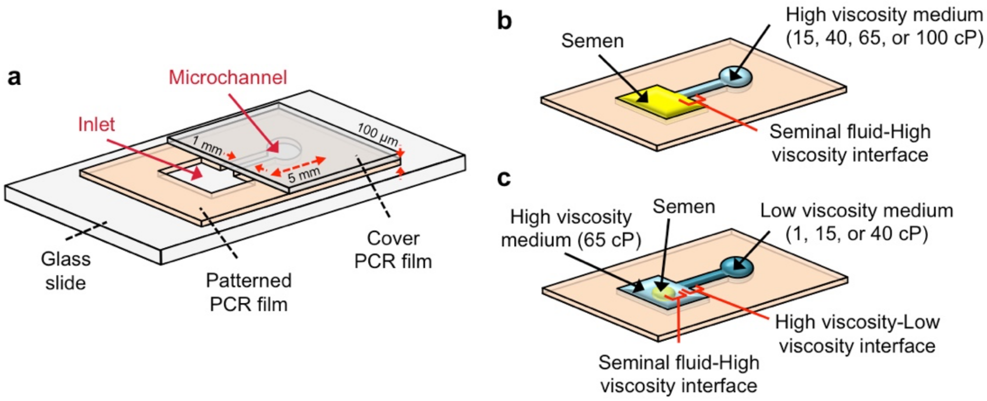

Supplementary Fig. 1 | Setup and medium conditions of the *in vitro* platform. a, Schematic of the *in vitro* platform, featuring a microchannel and inlet. **b**, Medium conditions of the platform that enable sperm group formation. The microchannel is filled with a high viscosity medium (15, 40, 65, or 100 cP) and the inlet is loaded with a semen sample, which results in an interface between the seminal fluid and the high viscosity medium. **c**, Medium conditions of the platform that enable the disbanding of sperm groups. The microchannel is filled with a lower viscosity medium (1, 15, or 40 cP) and the inlet is loaded with a 65 cP medium, resulting in a high-to-low viscosity interface. A droplet of semen is then carefully suspended in the 65 cP medium to establish an interface between the seminal fluid and the high viscosity medium.

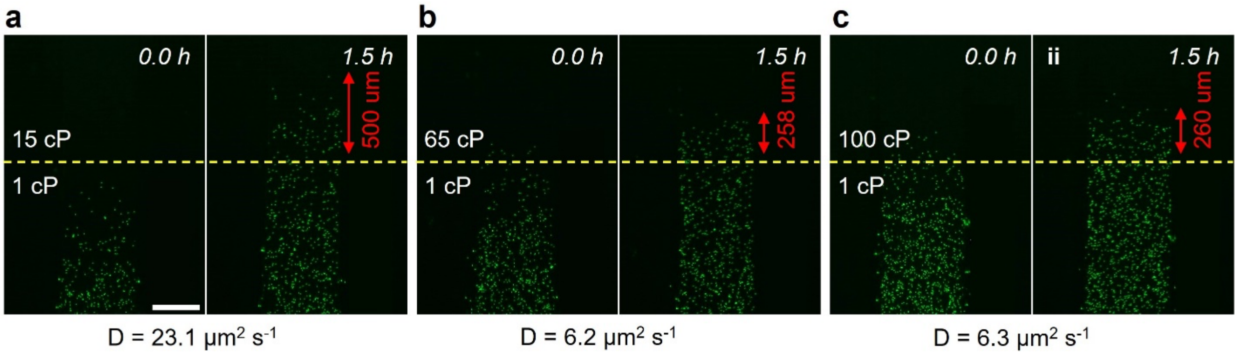

Supplementary Fig. 2 | Characterization of the laminar interface. a, The laminar interface between 1 cP (with fluorescent microbeads embedded) and 15 cP over 1.5 h. **b**, The laminar interface between 1 cP (with fluorescent microbeads embedded) and 65 cP over 1.5 h. **c**, The laminar interface between 1 cP (with fluorescent microbeads embedded) and 100 cP over 1.5 h. Scale bar: 1 mm.

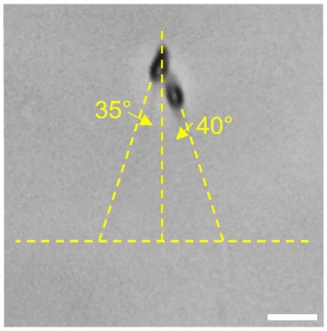

Supplementary Fig. 3 | The method at which sperm approach one another prior to attachment. Sperm approach one another at 35-40°, in a staggered configuration with one sperm ahead of the other. Scale bar: 5 µm.

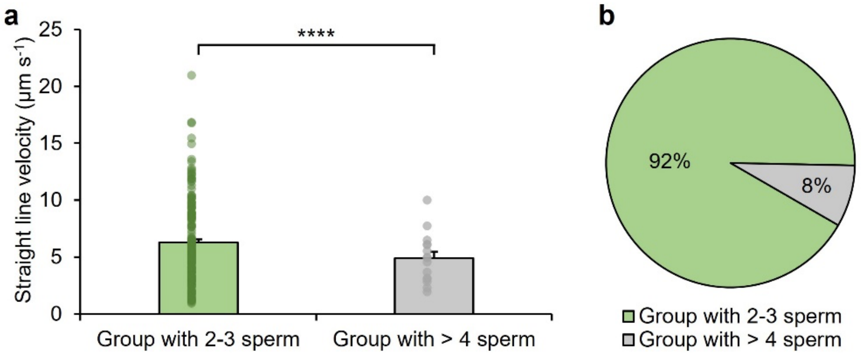

Supplementary Fig. 4 | Swimming velocity based on group size. a, Swimming velocity of sperm groups featuring 2-3 sperm in comparison to groups with more than 4 sperm (n = 5 independent replicates, N = 172 total sperm groups for 'Group with 2-3 sperm' and 35 total sperm groups for 'Group with > 4 sperm'; independent two-tailed Student's t-test). **b**, Breakdown of the sperm group sizes observed during analyses of velocity. Data are mean ± s.e.m. ****$P < 0.0001$.

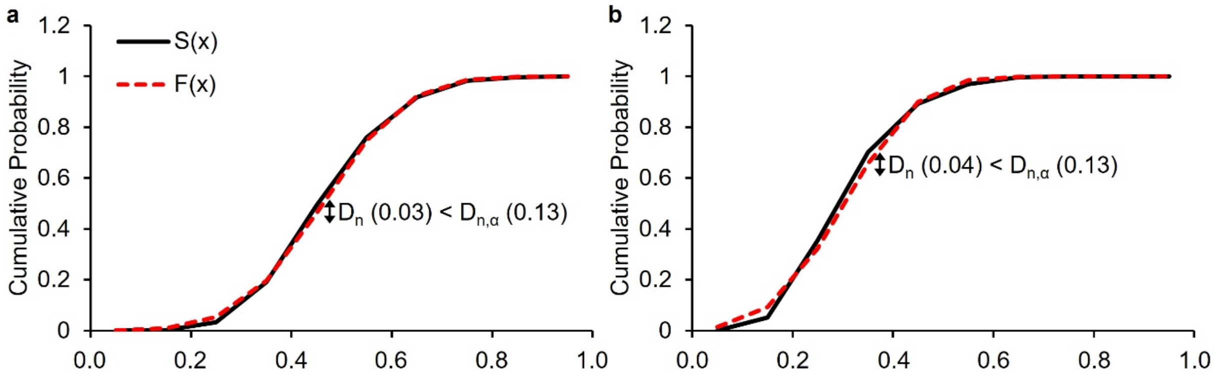

Supplementary Fig. 5 | The Kolmogorov-Smirnov Test. The Kolmogorov-Smirnov test for B/A distribution for **a**, sperm in a group and **b**, single sperm.

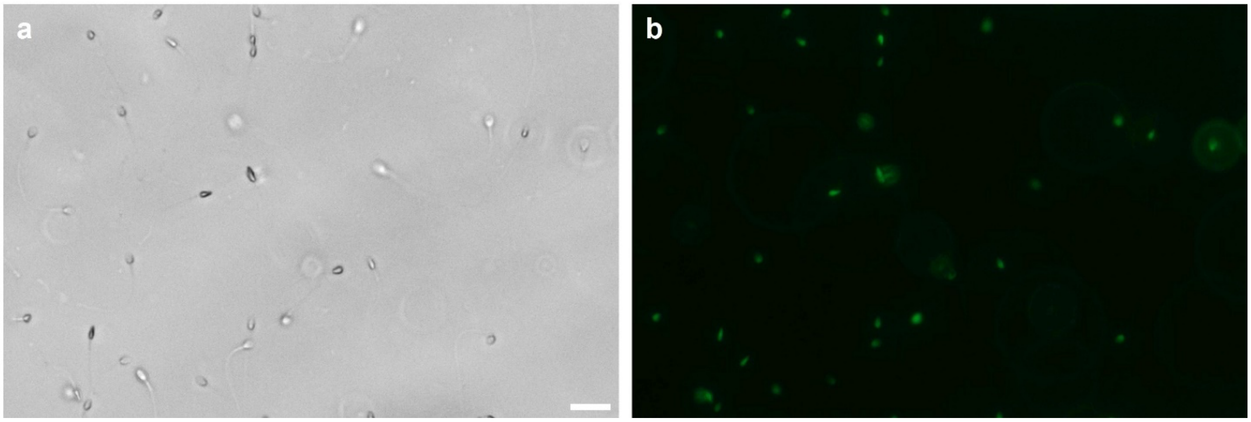

Supplementary Fig. 6 | Representative images of fluorescently tagged sperm from one part of the mixed sample. a, Representative grayscale image. **b**, Representative fluorescence image. Scale bar: 10 µm.

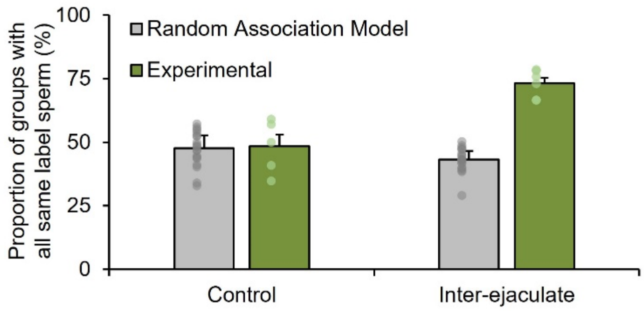

Supplementary Fig. 7 | Predicting preferential sperm cooperation using a random association model. Proportion of sperm groups with all same label sperm for control and inter-ejaculate tests, based on a statistically predicted random association model and experimental results. For the 'Random Association Model', 'Control': n = 20 independent test cases, N = 112 total sperm groups; and 'Inter-ejaculate': n = 20 independent test cases, N = 169 total sperm groups. For the 'Experimental', 'Control': n = 5 independent replicates, N = 112 total sperm groups; and 'Inter-ejaculate': n = 6 independent replicates, N = 169 total sperm groups. Data are mean ± s.e.m.
