# Supplementary material for: Human sperm cooperate to transit highly viscous regions on the competitive pathway to fertilization
Source: Commun Biol. 2023 May 6;6:495. doi: 10.1038/s42003-023-04875-2 (PMC10164193; doi:10.1038/s42003-023-04875-2)
Supplement: Supplementary file 2 — Description of Additional Supplementary Data [file 42003_2023_4875_MOESM2_ESM.pdf]

Description of Additional Supplementary Files

File name: Supplementary Data 1

Description: The source data of the charts in the manuscript.

File name: Supplementary Movie 1

Description: Sperm group formation in a 65 cP medium. The movie is at 2x speed.

File name: Supplementary Movie 2

Description: Sperm group consisting of 2 sperm in a 40 cP medium.

File name: Supplementary Movie 3

Description: Sperm group consisting of 3 sperm in a 65 cP medium.

File name: Supplementary Movie 4

Description: Sperm group consisting of 7 sperm in a 100 cP medium.

File name: Supplementary Movie 5

Description: Sperm group swimming in a methylcellulose medium of 100 cP.

File name: Supplementary Movie 6

Description: Sperm group swimming in a methylcellulose medium of 100 cP.

File name: Supplementary Movie 7

Description: Sperm group swimming in a methylcellulose medium of 100 cP.

File name: Supplementary Movie 8

Description: Sperm group migration between a 65 cP medium and a 15 cP medium without disbanding

File name: Supplementary Movie 9

Description: Sperm group migration between a 65 cP PVP medium and a 65 cP MC medium without disbanding.

File name: Supplementary Movie 10

Description: Sperm group migration between a 65 cP PVP medium and a 50 cP MC medium with disbanding.

File name: Supplementary Movie 11

Description: Sperm group migration between 65 cP PVP medium and the original seminal fluid with disbanding..

File name: Supplementary Movie 12

Description: Sperm group migration between 65 cP PVP medium and the original seminal fluid with disbanding.
