# Supplementary material for: Human sperm cooperate to transit highly viscous regions on the competitive pathway to fertilization
Source: Commun Biol. 2023 May 6;6:495. doi: 10.1038/s42003-023-04875-2 (PMC10164193; doi:10.1038/s42003-023-04875-2)
Supplement: Supplementary file 16 — Reporting Summary [file 42003_2023_4875_MOESM16_ESM.pdf]

nature portfolio

Corresponding author(s): David Sinton

Last updated by author(s): April 20, 2023

Reporting Summary

Nature Portfolio wishes to improve the reproducibility of the work that we publish. This form provides structure for consistency and transparency in reporting. For further information on Nature Portfolio policies, see our Editorial Policies and the Editorial Policy Checklist.

Statistics

For all statistical analyses, confirm that the following items are present in the figure legend, table legend, main text, or Methods section.

n/a	Confirmed	
☐	☒	The exact sample size (*n*) for each experimental group/condition, given as a discrete number and unit of measurement
☐	☒	A statement on whether measurements were taken from distinct samples or whether the same sample was measured repeatedly
☐	☒	The statistical test(s) used AND whether they are one- or two-sided<br>*Only common tests should be described solely by name; describe more complex techniques in the Methods section.*
☒	☐	A description of all covariates tested
☒	☐	A description of any assumptions or corrections, such as tests of normality and adjustment for multiple comparisons
☐	☒	A full description of the statistical parameters including central tendency (e.g. means) or other basic estimates (e.g. regression coefficient) AND variation (e.g. standard deviation) or associated estimates of uncertainty (e.g. confidence intervals)
☐	☒	For null hypothesis testing, the test statistic (e.g. *F*, *t*, *r*) with confidence intervals, effect sizes, degrees of freedom and *P* value noted<br>*Give P values as exact values whenever suitable.*
☒	☐	For Bayesian analysis, information on the choice of priors and Markov chain Monte Carlo settings
☒	☐	For hierarchical and complex designs, identification of the appropriate level for tests and full reporting of outcomes
☒	☐	Estimates of effect sizes (e.g. Cohen's *d*, Pearson's *r*), indicating how they were calculated

Our web collection on statistics for biologists contains articles on many of the points above.

Software and code

Policy information about availability of computer code

Data collection	The EVOS FL Auto Imaging software was used to acquire images. Bandicam was used to acquire videos.
Data analysis	ImageJ2/FIJI was used to analyze the images and videos (first converted to image sequences) as described in the Methods section. GraphPad Prism 7.0 was used to perform statistical analyses on the data.

For manuscripts utilizing custom algorithms or software that are central to the research but not yet described in published literature, software must be made available to editors and reviewers. We strongly encourage code deposition in a community repository (e.g. GitHub). See the Nature Portfolio guidelines for submitting code & software for further information.

Data

Policy information about availability of data

All manuscripts must include a data availability statement. This statement should provide the following information, where applicable:

- Accession codes, unique identifiers, or web links for publicly available datasets
- A description of any restrictions on data availability
- For clinical datasets or third party data, please ensure that the statement adheres to our policy

The main data supporting the results in this study are available within the paper and its Supplementary Information. The raw and analysed datasets generated during the study are available for research purposes from the corresponding author upon reasonable request.

Human research participants

Policy information about studies involving human research participants and Sex and Gender in Research.

Reporting on sex and gender	The findings apply to males. The sperm samples used in this study were purchased from ReproMed Ltd. sperm bank (Toronto, Canada) and obtained from Hannam Fertility Clinic (Toronto, Canada). Male sperm donors provided informed consent for research purposes to ReproMed Ltd. and Hannam Fertility and in accordance with the regulations of the Assisted Human Reproduction Act (approval number: 37629).
Population characteristics	The only information provided by ReproMed Ltd. and Hannam Fertility was a certificate acknowledging that all donor and patient sperm samples met the criteria for normozoospermia (i.e., the male is fertile) and passed the screening for pathogens.
Recruitment	Males undergoing fertility assessment donated a portion of their sperm sample to ReproMed Ltd. and Hannam Fertility for research purposes.
Ethics oversight	The University of Toronto Biosafety Office approved the experimental use of sperm samples (Assisted Human Reproduction Act, approval number: 37629).

Note that full information on the approval of the study protocol must also be provided in the manuscript.

Field-specific reporting

Please select the one below that is the best fit for your research. If you are not sure, read the appropriate sections before making your selection.

☒ Life sciences ☐ Behavioural & social sciences ☐ Ecological, evolutionary & environmental sciences

For a reference copy of the document with all sections, see nature.com/documents/nr-reporting-summary-flat.pdf

Life sciences study design

All studies must disclose on these points even when the disclosure is negative.

Sample size	For the majority of the experiments, the minimum replicate number eventually settled to be n = 5. The sample size corresponded to the total number of sperm appearing within or after the set testing time for each replicate experiment. We determined that the replicate numbers and sample sizes for each experiment were sufficient due to the minimal variability between values within n = 5 tests.
Data exclusions	No data were excluded from the analyses.
Replication	All attempts at replication were successful.
Randomization	All sperm samples met the criteria for normozoospermia and featured a concentration ranging between 20-25 million motile sperm/mL. The participants remained anonymous.
Blinding	Not applicable.

Reporting for specific materials, systems and methods

We require information from authors about some types of materials, experimental systems and methods used in many studies. Here, indicate whether each material, system or method listed is relevant to your study. If you are not sure if a list item applies to your research, read the appropriate section before selecting a response.

Materials & experimental systems

n/a	Involved in the study	
☒	☐	Antibodies
☒	☐	Eukaryotic cell lines
☒	☐	Palaeontology and archaeology
☒	☐	Animals and other organisms
☒	☐	Clinical data
☒	☐	Dual use research of concern

Methods

n/a	Involved in the study	
☒	☐	ChIP-seq
☒	☐	Flow cytometry
☒	☐	MRI-based neuroimaging
